# Swimming Characteristics of Bioinspired Helical Microswimmers Based on Soft Lotus-Root Fibers

**DOI:** 10.3390/mi8120349

**Published:** 2017-11-30

**Authors:** Jia Liu, Tiantian Xu, Yanming Guan, Xiaohui Yan, Chengwei Ye, Xinyu Wu

**Affiliations:** 1 Guangdong Provincial Key Laboratory of Robotics and Intelligent System, Shenzhen Institutes of Advanced Technology, Chinese Academy of Sciences, Shenzhen 518055, China; jia.liu1@siat.ac.cn (J.L.); ym.guan@siat.ac.cn (Y.G.); xy.wu@siat.ac.cn (X.W.); 2 CAS Key Laboratory of Human-Machine Intelligence-Synergy Systems, Shenzhen Institutes of Advanced Technology, Shenzhen 518055, China; 3 University of Chinese Academy of Sciences, Beijing 100049, China; 4 Department of Mechanical and Automation Engineering, The Chinese University of Hong Kong, Hong Kong, China; yanxh86@link.cuhk.edu.hk; 5 School of Science and Engineering, The Chinese University of Hong Kong, Shenzhen 518172, China; 116010256@link.cuhk.edu.cn

**Keywords:** helical microswimmer, soft fiber, spindle-like rotate, magnetic actuation

## Abstract

Various kinds of helical swimmers inspired by E. coli bacteria have been developed continually in many types of researches, but most of them are proposed by the rigid bodies. For the targeted drug delivery, the rigid body may hurt soft tissues of the working region with organs. Due to this problem, the biomedical applications of helical swimmers may be restricted. However, the helical microswimmers with the soft and deformable body are appropriate and highly adaptive in a confined environment. Thus, this paper presents a lotus-root-based helical microswimmer, which is fabricated by the fibers of lotus-root coated with magnetic nanoparticles to active under the magnetic fields. The helical microstructures are derived from the intrinsic biological structures of the fibers of the lotus-root. This paper aims to study the swimming characteristic of lotus-root-based microswimmers with deformable helical bodies. In the initial step under the uniform magnetic actuation, the helical microswimmers are bent lightly due to the heterogeneous distribution of the internal stress, and then they undergo a swimming motion which is a spindle-like rotation locomotion. Our experiments report that the microswimmers with soft bodies can locomote faster than those with rigid bodies. Moreover, we also find that the curvature of the shape decreases as a function of actuating field frequency which is related to the deformability of lotus-root fibers.

## 1. Introduction

Artificial micro-/nanorobots have attracted lots of researchers to carry on extensive study due to their considerable promise for diverse biomedical tasks such as targeted therapy [1,2,3,4,5,6,7,8,9], tissue removal [10,11] and micro-manipulation [12,13,14,15]. Purcell found the advantages of the nonreciprocal motion and demonstrated two efficient swimming modes at low Reynolds number [16,17]: the flexible oar and the corkscrew. Although Qiu et al. [18] presented a microswimmer that moves with reciprocal periodic body-shape changes in non-Newtonian fluids, its propulsion performance depended on the fluid viscosity upon varying the shear rate. Recently, more attention has been focus on helical magnetic microswimmers which can convert the rotation energy along their helical axis into the translation energy actuated by the uniform magnetic field [2]. Inspired by mastigoneme structures in nature, the research group from Swiss Federal Institute of Technology Zurich (ETH Zurich) fabricated an artificial helical microswimmers with multiple flagella and mastigonemes, modeled its propulsion model and studied the relation between the length and the velocity [19]. Tabak et al. [20] presented the 6-DOF kinematic models for two-link helical micro/nanoswimmers with the aid of computational fluid dynamics (CFD) analysis. Erman et al. [21] presented three-dimensional trajectory following by means of resistive force theory (RFT) for a rigid helical microswimmer. Nourmohammadi et al. [22] proposed a 3-DOF swimming microrobot with three helical flagella and its dynamical model. Mahoney et al. [23] developed a full 6-DOF model for helical microswimmer and directly controlled its spatial velocity instead of its pitch and rotation speed. Xu et al. [24] analyzed the propulsion characteristic of scaled-up helical microswimmers with different magnetic head or magnetic tail and determined the propulsion matrice. Then Xu et al. [25] accomplished the planar path following based on a 3-D steering scaled-up helical microswimmers. Zhang et al. [26] reported artificial bacterial flagella (ABF) that can swim in a controllable fashion using weak magnetic fields and also analyzed the manipulated performance manipulated by the thrust force. Tottori et al. [27] presented the assembled structure exhibiting the different swimming properties, its dynamic behavior may guide to design active suspensions for drug delivery and imaging. Schamel et al. [28] presented helical nano-propellers showing significant advantages in viscoelastic hyaluronan gels, such as faster or notable propulsion. Walker et al. [29] experimentally analyzed the optimal helix length by trading off between the maximal net chirality and the minimal vicious friction. Similarly, Xu et al. [30] carried on the extended experiment to analysis the four factors ( pitch, turn, width, thickness). In all, these researches focused on the swimming characteristics for the rigid body of the helical microswimmer.

Qiu et al. [31] used 3D laser direct writing tool and e-beam deposition methods to manufacture microswimmers with 5 μm-diameter and 16 μm-length. Ghosh et al. [32] employed the shadow-growth method to fabricate the glass (SiO${}_{2}$) nanostructured propellers. Qiu et al. [33] fabricated the helical bodies of ABFs from a available biocompatible photoresist OrmoComp (micro resist technology GmbH, Berlin, Germany), and coated them with Fe. Xu et al. [34] present a swimmer with belt-like soft tail made of polydimethylsiloxane (PDMS). Stanton et al. [35] employed the electrochemical method to fabricate the Ppy microtubes. Li et al. [36] initially used anodic aluminum oxide (AAO) membrane templates with pore diameter of 200 nm for preparing the helical nanoswimmers. In addition, Li et al. [37] employed the template-assisted method to fabricate the bisegment magneto-acoustic hybrid nanomotors. It is a challenge to product the three-dimensional helical micro/nanostructures in a large scale which involves either custom or novel fabrication process, including the optical lithography, two-photon stereo-lithography, self-folding thin-films, micro/nanomachining, micro/nanoimprinting, and micro/nanomolding technique [38]. These fabrication processes can produce a variety of the advanced applications, but they only take place in the laboratory with the specific instrument. Moreover, material used in the fabrication should be biocompatible with respect to in vivo biomedical applications.

In the nature, there are a diversity of microstructures such as plant fibers. Gao et al. [39] proposed plant-based helical microstructures derived from Xylem vessels covered with a thin Ti and Ni layer directly on the spiral vessels, and adopted the propulsion mechanism with respect to the rigidly helical structure, but not described the swimming characteristic in detail. Yan et al. [40] chose the cyanobacterium Spirulina as a based structure to demonstrate the bio-templated synthesis with three steps: precursor deposition, annealing treatment and reduction processing. Employing the physically synthetic methods, we can transform the structures of the plant fiber into the functional microstructures which utilizes their intrinsic natural morphology. Hence, we demonstrate the lotus-root-based magnetically propelled helical microswimmers. The novel helical microswimmers are fabricated by simply coating the lotus-root-based fibers with a thin magnetic layer. Moreover, the lotus-root-based magnetically propelled helical microswimmers can be coated with superparamagnetic nano-particles with manipulated magnetic anisotropy.

The main contributions of this papers are the following: (1) demonstrate the soft and deformable helical microswimmers based on the fibre of the lotus-root; (2) find the spindle-like rotation locomotion; (3) investigate the spindle-like rotation locomotion characteristic.

## 2. Swimming Characteristic of the Helical Microswimmers

### 2.1. Theoretical Analysis of Rigid Helical Microswimmers

Purcell suggested that every motion is linear at low Reynolds number, so the propulsion matrix is mapped by the force and torque with the constant coefficient [16]. At low Reynolds number regime, a simplified Stokes equation governs hydrodynamics. An external force applied on the helix that translates along its axis can make it necessarily rotate. An external torque applied on the helix that rotates along its axis also can make it necessarily translate.

Generally, the resistive-force theory (RFT) can calculate the thrust force and torque by the helical motion. The underlying assumption of the intuitive approach is that the hydrodynamic force is proportional to the local body velocity with the constant resistance coefficients (RC) which are derived from the slender body theory. The theory approximates a slender filament by a line distribution of singularity solutions [9].

A helical microswimmer in the fluid at low Reynolds number which is Re=ρulρulμμ, and the fluid mechanics can be presented by the Stokes equations

(1) $$\left\{\begin{array}{c} \nabla p=\mu \nabla^{2}u\\ \nabla u=0 \end{array}\right.$$

where ρ and μ are the density and dynamic viscosity of the fluid, respectively. *u* and *l* are the velocity of the objective and characteristic length, respectively. *p* is the pressure. There is no time-dependent terms in this equation, hinting that the resultant propulsion force only depends on the propeller relative position. Therefore, the microswimmers have to employ non-reciprocal mode in order to locomote forward.

The external force and torque acting on the microswimmer can be modeled by the magnetic force, $f_{m}$ and $\tau_{m}$, respectively. The resistive friction force and torque can be presented by $f_{c}$ and $\tau_{c}$, respectively. The viscous drag force and torque can be expressed by $f_{d}$ and $\tau_{d}$, respectively. With the above analysis, the dynamic equations are given by

(2) $$\left\{\begin{array}{c} \tau_{d}+\tau_{c}+\tau_{m}=0\\ f_{d}+f_{c}+f_{m}=0 \end{array}\right.$$

**Figure 1:**
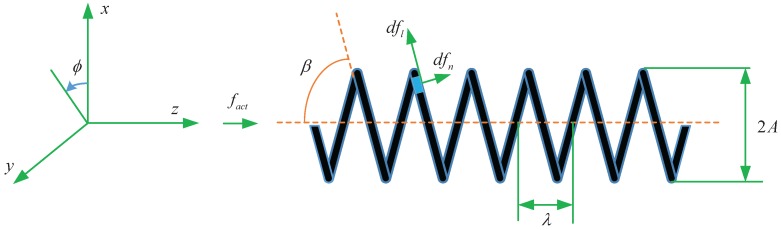
Schematic of the helical microswimmer.

When a magnetic field with frequency $f_{act}$ is applied on the magnetized lotus-root-based helical microswimmer. Figure 1 shows the schematic of the helical micro-swimmer and the vicious force $f_{d}$ and vicious torque $\tau_{d}$ acting on the body due to helical propulsion.

For an element ds on the helix, the propulsive force in *z*-axis direction, in terms of its normal component $df_{n}$ and longitudinal component $df_{l}$, is

(3) $$df_{d}=df_{n}\sin\beta -df_{l}\cos\beta$$

The force $df_{\phi }$ in ϕ direction is equal to

(4) $$df_{\phi }=-df_{n}\cos\beta -df_{l}\sin\beta$$

which results in the vicious torque $d\tau_{d}$ along *z*-axis

(5) $$d\tau_{d}=Adf_{\phi }=-Adf_{n}\cos\beta -Adf_{l}\sin\beta$$

where *A* is the amplitude of the helix and β is the constant pitch angle between the helix and the *z*-axis.

(6) $$\tan\beta =\frac{2\pi }{\lambda }A$$

where λ is the wavelength of the helix.

The velocity vector of the element ds is constructed through the two components of $v_{n}$ and $v_{l}$ which arise from the forward movement *v* and angular movement $Af_{act}$

(7) $$\left\{\begin{array}{c} v_{n}=v\sin\beta -Af_{act}\cos\beta \\ v_{l}=-v\cos\beta -Af_{act}\sin\beta \end{array}\right.$$

Hancock et al. [41] proposed theoretical analysis to calculate the normal coefficient $C_{n}$ and tangential coefficient $C_{l}$ of viscous resistance on the thin cylindrical filament swimming in a viscous fluid.

(8) $$\left\{\begin{array}{c} df_{n}=-C_{n}v_{n}ds\\ df_{l}=-C_{l}v_{l}ds \end{array}\right.$$

The length of segment ds can be rewritten as ds=dzdzcosβcosβ. $C_{n}$ and $C_{l}$ are the corresponding coefficients of resistance driven empirically by Johnson and Brokaw [42]

(9) Cn=4πμln2λ2λdd+0.5Cl=2πμln2λ2λdd−0.5

Therefore, substituting (7) into (8), which yields,

(10) $$\left\{\begin{array}{c} df_{n}=C_{n}\left(-v\sin\beta +Af_{act}\cos\beta \right)\sec\beta dz\\ df_{l}=C_{l}\left(v\cos\beta +Af_{act}\sin\beta \right)\sec\beta dz \end{array}\right.$$

The total propulsive force $f_{d}^{i}$ and torque $\tau_{d}^{i}$ on one pitch of the helix can be expressed by integrating the force and torque on the segment ds along *z*-axis.

(11) $$\left\{\begin{array}{c} f_{d}^{i}=\int_{z=0}^{z=\lambda }df_{d}\\ \tau_{d}^{i}=\int_{z=0}^{z=\lambda }d\tau_{d} \end{array}\right.$$

where *i* is the *i*th pitch.

### 2.2. Analysis on Spindle-Like Rotation Locomotion Steps

**Figure 2:**
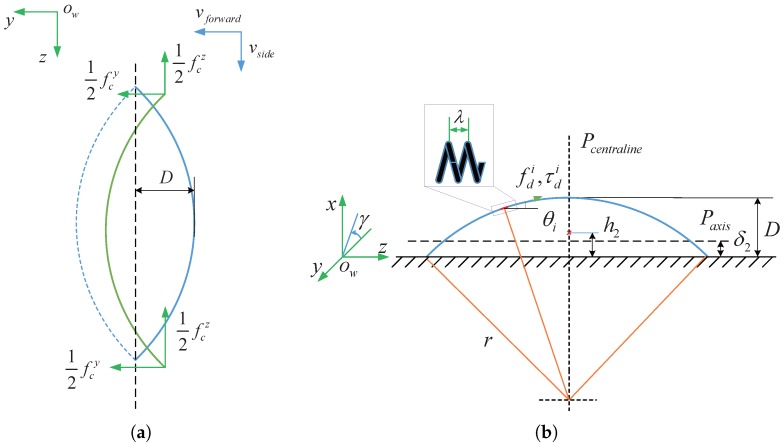

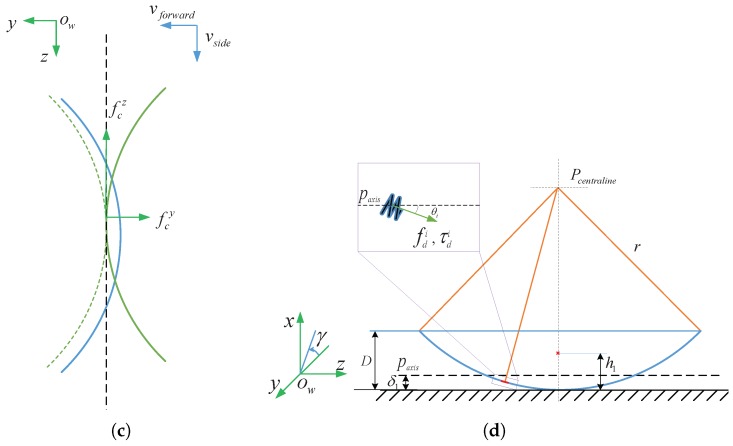
Spindle-like rotation locomotion: Schematic of the two-point-support step (**a**) Spinning $180^{\circ }$ along *z*-axis; (**b**) Schematic of the total propulsive force $f_{d}^{i}$ and torque $\tau_{d}^{i}$ on one pitch of the helix, $h_{2}$ is the center height from the center position to the bottom; Schematic of the one-point-support step (**c**) Spinning $180^{\circ }$ along *z*-axis; (**d**) Schematic of the component propulsive force $f_{d}^{i}$ and torque $\tau_{d}^{i}$ on one pitch of the helix, $h_{1}$ is the center height from the center position to the bottom.

Due to advantage of their intrinsic natural morphology, some synthetic technology can be employed to transform highly structured plant fiber into functional materials. We first utilize the lotus-root fiber to fabricate the propelled helical microswimmers. To obtain stretched spiral fibres, lotus-roots were gently cut, and the two segments were pulled apart to a fixed distance, and then the helixes can be got. The new helical microswimmers are fabricated by the simple coating of lotus-root-based fibers with a thin magnetic layer. Specifically, the lotus-root-based magnetically propelled helical microswimmers can be coated with superparamagnetic nano-particles with manipulated magnetic anisotropy. We adjust the magnetic axis to be approximately perpendicular to the axis of the helical microswimmer. Due to the heterogeneous distribution of the shrinking stress, the magnetically propelled helical microswimmers are bent lightly initially for different length. Its shaping principle is similar to the strain engineering which is a widely used technique [43]. During the named spindle-like rotation locomotion shown in Figure 2a–d, the microswimmers can locomote with a little sliding actuated by uniform magnetic fields.

The open-loop control of the propulsion of lotus-root microswimmer is achieved by employing a uniform rotating magnetic field. Figure 2a,b shows the two-point-support rotation locomotion step. Within one step period, the two ends of the helical microswimmers touch the bottom of the container and support the whole body to spin $180^{\circ }$. This step is followed by another, as is shown in Figure 2c,d. The apex of the deformable helical body touches the bottom of the container and supports the whole body to spin another $180^{\circ }$. Repeating the process can create the spindle-like rotation locomotion which is different from the rigid rotation locomotion. This spindle-like rotation can both push the helical body to locomote forward perpendicular to $p_{axis}$ and push the helical body side-slide a little bit along $p_{axis}$. Figure 2 illustrates that $v_{side}$ is parallel to $p_{axis}$, $v_{forward}$ is perpendicular to $p_{axis}$ .

**Figure 3:**
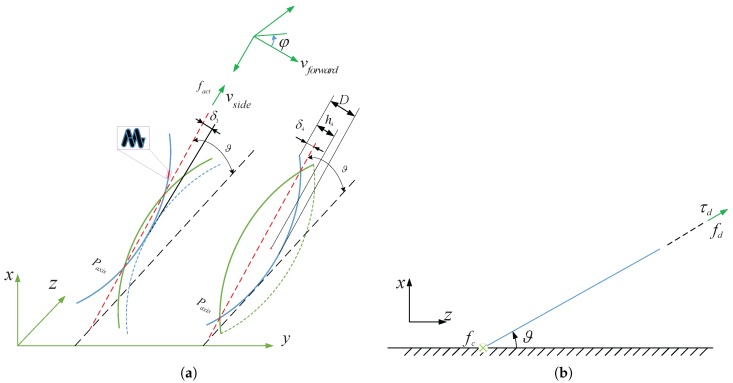
Schematic of spindle-like rotation locomotion with an inclination angle ϑ (**a**) two steps for spindle-like rotation locomotion cycle from top view: zero-point-support step (the left one) and one-point-support step (the right one); (**b**) Schematic of propulsive force $f_{d}$, the propulsive torque $\tau_{d}$, and the friction force $f_{c}$ from side view.

In another experiment, Figure 3 show the spindle-like rotation locomotion with an inclination angle ϑ but one-terminal of the lotus-root microswimmer touches the bottom of the container. The left one in Figure 3a shows the one-point-support step. Within the step, the one end of the helical microswimmers touches the bottom of the container and supports the whole body to spin $180^{\circ }$. Then for another step, the right one in Figure 3a shows that the one-terminal of the deformable helical body aparts from the bottom of the container and the whole body suspends in liquid spinning another $180^{\circ }$. Repeating the process can create spindle-like rotation locomotion with an inclination angle which can make the helical body to locomote forward perpendicular to $p_{axis}$.

### 2.3. Theoretical Analysis of Soft Helical Microswimmers

In order to simplify the dynamic model, we make the assumption that its structure is symmetric about $p_{centraline}$, the following expression along $p_{centraline}$ perpendicular to the instantaneous axis $p_{axis}$ in Figure 2b can be given,

$$\sum_{i=1}^{n}f_{x}^{i}\sin\theta_{i}=0$$

where $f_{x}^{i}$ is the component of the $f_{d}^{i}$ along *x*-axis in Figure 2b.

Therefore, the following expression along the instantaneous axis $p_{axis}$ in Figure 2b can be given,

(12) $$\left\{\begin{array}{c} \tau_{d}=\sum_{i=1}^{n}\tau_{d}^{i}\cos\theta_{i}\\ f_{d}=\sum_{i=1}^{n}f_{d}^{i}\cos\theta_{i} \end{array}\right.$$

where *n* denotes the number of the pitch in the whole body, $f_{d}^{i}$ and $\tau_{d}^{i}$ can be obtained from (11),

$$f_{d}^{i}=-\lambda \left(C_{n}\sin\beta \tan\beta +C_{l}\cos\beta \right)v+\lambda A\sin\beta \left(C_{n}-C_{l}\right)f_{act}$$

$$\tau_{d}^{i}=\lambda A\sin\beta \left(C_{n}-C_{l}\right)v-\lambda A^{2}\left(C_{n}\cos\beta -C_{l}\sin\beta \tan\beta \right)f_{act}$$

The resistive friction force and torque can be used to model the interactive relation between the robot and the environment. For one step in spindle-like rotation locomotion, the ends of the helical microswimmers touches the bottom of the container and supports the whole body to spin $180^{\circ }$. A simple Coulomb friction model is employed in Figure 2a, which yields

$$\left\{\begin{array}{c} f_{c}^{z}=\mu_{z}\left(\rho_{lotus}-\rho_{liquid}\right)V_{lotus}g\\ f_{c}^{y}=\mu_{y}\left(\rho_{lotus}-\rho_{liquid}\right)V_{lotus}g \end{array}\right.$$

where $\mu_{z}$ and $\mu_{y}$ are the friction constants, $\left(\rho_{lotus}-\rho_{liquid}\right)V_{lotus}$ is the total net mass, *g* is the gravitational constant.

The resultant resistive torque $\tau_{c2}$ in Figure 2a,b can be expressed as,

(13) $$\tau_{c2}=2\int_{0}^{\frac{\pi }{2}}\delta_{2}f_{c}^{y}\sin\gamma d\gamma$$

where $\delta_{2}$ represents the distance from the contact point to the instantaneous axis $p_{axis}$, γ represents the angle from the y-z plane to the helix plane containing the helical body.

In another step in Figure 2c,d where the apex of the deformable helical body touches the bottom of the container, assuming that there is a constant sliding friction coefficient, the resistive friction force can also be expressed as $f_{c}^{z}$ and $f_{c}^{y}$. whereas, the distance from the contact point to the instantaneous axis $p_{axis}$ is expressed as $\delta_{1}$, which yields,

(14) $$\tau_{c1}=2\int_{0}^{\frac{\pi }{2}}\delta_{1}f_{c}^{y}\sin\gamma d\gamma$$

Within the two steps in spindle-like rotation locomotion, the total friction resistive torque $\tau_{c}$ can be expressed as,

(15) $$\tau_{c}=\tau_{c1}+\tau_{c2}$$

The total magnetic force ${\vec{f}}_{m}$ and torque ${\vec{\tau }}_{m}$ acting on the body can be represented as

(16) $$\left\{\begin{array}{c} {\vec{\tau }}_{m}=M\times B\\ {\vec{f}}_{m}=\left(M\cdot \nabla \right)B \end{array}\right.$$

where *M* is the magnetization vector of the magnetic particle, and *B* is the magnetic field vector.

The magnetic coils in our lab can only create an uniform rotating magnetic field, so the gradient of the magnetic field is zero and the magnetic force $f_{m}$ becomes zero. We focus on the dynamic analysis that the helical microswimmer can perform spindle-like rotation locomotion approximately in sync with rotating magnetic field at low actuate frequency. In conclusion, submitting (13), (15) and (16) into (2), and the final dynamic equations can be given by,

(17) $$\left[\begin{array}{cc} a_{11} & a_{12}\\ a_{21} & a_{22} \end{array}\right]\left[\begin{array}{c} v\\ f_{act} \end{array}\right]=\left[\begin{array}{c} c_{1}\\ c_{2} \end{array}\right]$$

where these following coefficients of the dynamic model can be given by,

$$a_{11}=\sum_{i=1}^{n}\lambda A\sin\beta \left(C_{n}-C_{l}\right)\cos\theta_{i}$$

$$a_{12}=-\sum_{i=1}^{n}\lambda A^{2}\left(C_{n}\cos\beta -C_{l}\sin\beta \tan\beta \right)\cos\theta_{i}$$

$$a_{21}=-\sum_{i=1}^{n}\lambda \left(C_{n}\sin\beta \tan\beta +C_{l}\cos\beta \right)\cos\theta_{i}$$

$$a_{22}=\sum_{i=1}^{n}\lambda A\sin\beta \left(C_{n}-C_{l}\right)\cos\theta_{i}$$

$$c_{1}=\left(\rho_{lotus}-\rho_{liquid}\right)V_{lotus}g\left(2\int_{0}^{\frac{\pi }{2}}\delta_{2}f_{c}^{y}\sin\gamma d\gamma +2\int_{0}^{\frac{\pi }{2}}\delta_{1}f_{c}^{y}\sin\gamma d\gamma \right)-MB$$

$$c_{2}=f_{c}^{z}$$

Then we can get the following equations, which characterized the process of the lotus-root microswimmer spindle-like rotation locomotion on the bottom of the container. Within one locomotion cycle (γ changes from $0^{\circ }$ to $360^{\circ }$), the relation between locomotion velocity $v_{forward}$ perpendicular to $P_{axis}$ axis, locomotion velocity $v_{side}$ along $P_{axis}$ axis and frequency is established from (17).

(18) $$f_{act}^{1}=\frac{c_{1}a_{21}-c_{2}a_{11}}{a_{12}a_{21}-a_{22}a_{11}}$$

(19) $$v_{side}=\frac{c_{1}a_{22}-c_{2}a_{12}}{a_{22}a_{11}-a_{12}a_{21}}$$

(20) $$v_{forward}=\frac{1}{2}\left[\left(h_{1}-\delta_{1}\right)f_{act}^{1}+\left(h_{2}-\delta_{2}\right)f_{act}^{1}\right]$$

For a rigid helical body, its locomotion velocity is $f_{act}A$ in Figure 1. Because of $\frac{1}{2}\left[\left(h_{1}-\delta_{1}\right)+\left(h_{2}-\delta_{2}\right)\right]>A$, it can be inferred that $v_{forward}>f_{act}A$. Figure 3a shows spindle-like rotation locomotion with an inclination angle ϑ in each step from top view, the left one is one-point-support step, the right one is the zero-point-support step. Figure 3b shows the propulsion force, the propulsion force and the friction force from side view. Experiments find that the helical body is not propelled apart from the bottom of the container. So we can conclude that $v_{side}=0$ since the propulsion force $f_{d}\sin\vartheta$ is less than the net gravitational force $\left(\rho_{lotus}-\rho_{liquid}\right)V_{lotus}g$.

Based on above discussions, a simplified torque balance equation can be derived in one-point-support step for spindle-like rotation locomotion with an inclination angle ϑ (the right one in Figure 3a),

(21) $$\begin{array}{c} \sum_{i=1}^{n}(-\lambda A^{2}\left(C_{n}\cos\beta -C_{l}\sin\beta \tan\beta \right)f_{act}^{4}\cos\theta_{i})+MB-\\ 2\int_{0}^{\frac{\pi }{2}}f_{c}\delta_{3}\cos\phi d\phi -2\int_{0}^{\frac{\pi }{2}}\mu \left(\rho_{lotus}-\rho_{liquid}\right)V_{lotus}g\left(h_{4}-\delta_{4}\right)\sin\varphi d\phi =0 \end{array}$$

where $\mu_{fric}$ is the friction coefficient.

$$f_{c}=\mu_{fric}\left(\left(\rho_{lotus}-\rho_{liquid}\right)V_{lotus}g-f_{d}\sin\vartheta \right)$$

$$f_{d}=\sum_{i=1}^{n}(\lambda A\sin\beta \left(C_{n}-C_{l}\right)f_{act}\cos\theta_{i})$$

In another step, a simplified torque balance equation can be derived in zero-point-support step for spindle-like rotation locomotion with an inclination angle ϑ (the left one in Figure 3a),

(22) $$\begin{array}{c} \sum_{i=1}^{n}(-\lambda A^{2}\left(C_{n}\cos\beta -C_{l}\sin\beta \tan\beta \right)f_{act}^{3}\cos\theta_{i})+MB-\\ 2\int_{0}^{\frac{\pi }{2}}\mu \left(\rho_{lotus}-\rho_{liquid}\right)V_{lotus}g\left(h_{4}-\delta_{3}\right)\sin\varphi d\phi =0 \end{array}$$

Then we can get the following equations, which characterized the process of the lotus-root microswimmer spindle-like rotation locomotion with an inclination angle ϑ. $f_{act}^{4}$ is derived from (21), $f_{act}^{3}$ is derived from (22). Within one locomotion cycle (φ changes from $0^{\circ }$ to $360^{\circ }$), the relation between spindle-like rotation locomotion velocity $v_{forward}^{i}$ perpendicular to $P_{axis}$ axis and frequency is established.

(23) $$f_{act}^{4}=\frac{MB-2\int_{0}^{\frac{\pi }{2}}f_{c}\delta_{3}\cos\phi d\phi -2\int_{0}^{\frac{\pi }{2}}\mu \left(\rho_{lotus}-\rho_{liquid}\right)V_{lotus}g\left(h_{4}-\delta_{4}\right)\sin\varphi d\phi }{\sum_{i=1}^{n}(\lambda A^{2}\left(C_{n}\cos\beta -C_{l}\sin\beta \tan\beta \right)\cos\theta_{i})}$$

(24) $$f_{act}^{3}=\frac{MB-2\int_{0}^{\frac{\pi }{2}}\mu \left(\rho_{lotus}-\rho_{liquid}\right)V_{lotus}g\left(h_{4}-\delta_{3}\right)\sin\varphi d\phi }{\sum_{i=1}^{n}(\lambda A^{2}\left(C_{n}\cos\beta -C_{l}\sin\beta \tan\beta \right)\cos\theta_{i})}$$

(25) $$v_{forward}^{\vartheta }=\frac{1}{2}\left[\left(h_{4}-\delta_{3}\right)f_{act}^{3}+\left(h_{4}-\delta_{4}\right)f_{act}^{4}\right]$$

where $h_{4}$ is the center height from the center position to the end of the helical body, $\delta_{3}$ is the distance between the apex and the instantaneous axis $P_{axis}$, $\delta_{4}$ is the distance between the end and the instantaneous axis $P_{axis}$. Due to its whole body suspending in liquid for $f_{act}^{3}$, its locomotion velocity $\left(h_{4}-\delta_{3}\right)f_{act}^{3}=0$. For a rigid helical body, its spindle-like rotation locomotion velocity is $f_{act}A$ in Figure 1. Because of $\frac{1}{2}\left(h_{4}-\delta_{4}\right)>A$, it can be inferred that $v_{forward}^{\vartheta }>f_{act}A$.

## 3. Experimental Setup and Fabrication of the Helical Microswimmers

**Figure 4:**
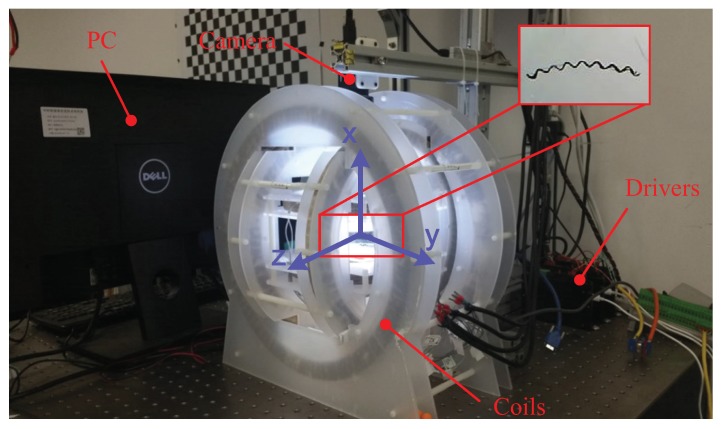
3D Helmholtz coil system.

The helical microswimmers in this paper are actuated by a 3D Helmholtz coil system shown in Figure 4. The 3D Helmholtz coil system can generate a uniform magnetic field in working space with size of approximately 80×50×40 mm, drived by 3 Maxon ESCON 70/10 motor drivers (Maxon Motor, Sachseln, Switzerland). PC can send out control signals through Sensoray S826 PCIe A/D IO card to the motor drivers. This system also comes with a single camera (PointGreyGS3-U3-41C6M, FLIR Integrated Imaging Solutions, Inc., Richmond, BC, Canada) mounted on the top of the 3D Helmholtz coils, providing overviews for monitoring. In the experiments, the rotations of the the helical microswimmers can be recorded by the camera. The frame rate is about 50 frames/s. The spindle-like locomotion velocity is measured offline by the traveled distance in pixel per unit time. Then, it is converted into international unit.

**Figure 5:**
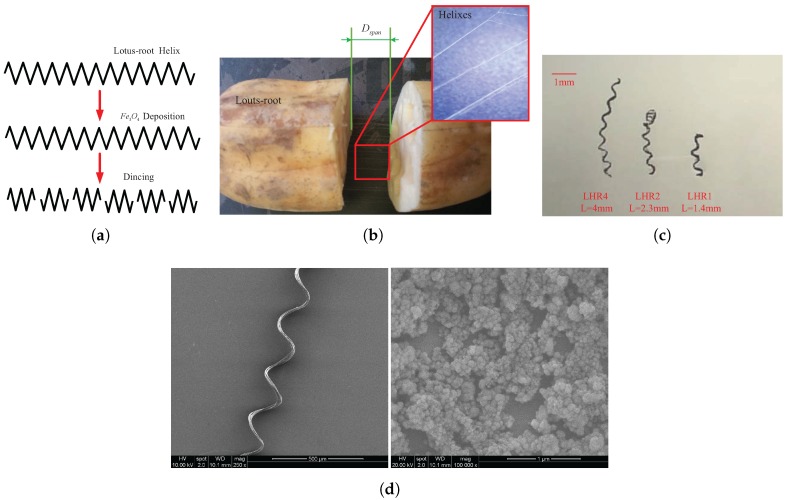
(**a**) Schematic illustrating the steps for the preparation of the lotus-based microswimmers; (**b**) Image illustrating arbitrary stretching of the lotus-root and the isolated helical structure; (**c**) Image of multiple magnetic helical microswimmers from (**a**); (**d**) The left: low-magnification scanning electron microscope (SEM) image of the lotus-root’s fiber with magnetic coating. The right: enlarged SEM image of the selected area in the left, showing a relatively-uniform magnetic coating. Field emission scanning electron microscope (FESEM) images were acquired using a FEI Quanta 400F (FEI Company, Hillsboro, OR, USA) microscope with an accelerating voltage of 10 kV.

The swimming behaviour of the deformable helical microswimmers has not been clearly defined mainly due to the limited fabrication. Moreover, the traditional fabrication process is time-consuming. That is why scaled-up helical swimmers are designed. A simple reproducible method is illustrated in Figure 5a. The helical fibers are mechanically isolated from the roots of lotus. The whole process goes sequentially through cutting, breaking and stretching. We can controll the streching span $D_{span}$ in Figure 5b and thus tailor the length of the helixes. To enable magnetic acutation, the obtained helixes are subject to the coating of magnetic materials. Then they are diced into the desired lengths. The helical dimensions of the microswimmers, and the overall shape, remain constant throughout the whole fabrication process. The high density of helixes within the lotus-root in Figure 5b provides an ideal platform for easy production of the functional helical microswimmers in Figure 5c. This method is thus extremely promising because it provides a cost-effective and straightforward production of helical magnetic microswimmers and offers substantial savings in material requirements and processing costs compared to the commonly used methods for fabricating helical microswimmers. Employing above methods, we can fabricate three helical microswimmers with different length including 4 mm, 2.3 mm, 1.4 mm and name them LRH4, LRH2 and LRH1 respectively in Figure 5c. Based on the following scanning electron microscopy (SEM) image in Figure 5d, the distribution of magnetic matter (i.e., Fe${}_{3}$O${}_{4}$) is relatively-uniform on lotus-root’s fiber.

The fluid motion around the helical swimmer can be characterized by one dimensionless parameter: the Reynolds numbers. Due to the small scale of E. coli bacteria, its swimming environment can be characterized by low Reynolds numbers. Consequently we select the 12.5% glycerinum solution to simulate the low Reynolds numbers environment. As for the helical microswimmers swimming in the glycerol solution, its density and viscosity in 20 ${}^{\circ }$C are 1.033 g/cm${}^{3}$ and 13 mPa·s, respectively. The locomotion velocity of the helical swimmer is 2–7 mm/s. Therefore, the calculated Reynolds number is approximately 0.16–0.55, the helical swimmer locomotes at low Reynolds numbers.

Here we use element analysis to evaluate the amount of superparamagnetic nanoparticles on a lotus-root’s fiber with an energy-dispersive X-ray spectroscopy (EDX) analyzer mounted on the FEI Quanta 400F(FEI Company, Hillsboro, OR, USA) (accelerating voltage at 20 kV). The Fe element is assigned to magnetic coating (Fe${}_{3}$O${}_{4}$), C element to the lotus-root’s fiber, O element to the magnetic coating and lotus-root’s fiber, and Si element to the silicon subtract used to support scanning electron microscope (SEM) samples. According to these data from the EDX analyzer, magnetic coating ratio ψ=mhelixmhelixmcoatingmcoating takes about 5.4%, $m_{helix}$ denotes the total mass of the lotus-root’s fiber and the magnetic coating layer, $m_{coating}$ denotes the mass of the magnetic coating layer.

## 4. Results and Discussion

### 4.1. Experiments and Analysis on Spindle-like Rotation Locomotion

The following experiment were carried out to verify the curvature of the helical body under different actuate frequency as well as the relation between the spindle-like locomotion velocity $v_{forward}$, the propulsion velocity $v_{side}$ and the actuate frequency $f_{act}$. To quantify the curvature, we define the distance D between the apex of the deformable helical body and the end shown in Figure 2a.

**Figure 6:**
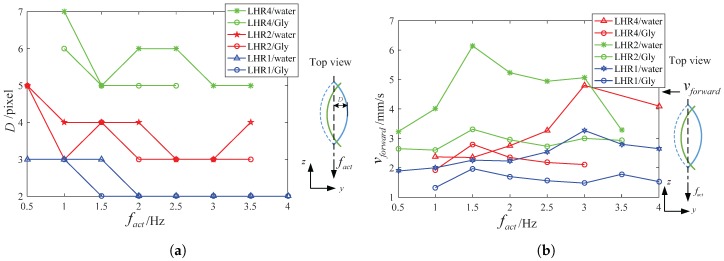
(**a**) The relation between distance D and actuate frequency $f_{act}$ for different length; (**b**) The relation between spindle-like locomotion velocity $v_{forward}$ and actuate frequency $f_{act}$ for different length.

Figure 6a illustrates the distance D for LRH4, LRH2, LRH1 in function of the actuate frequency $f_{act}$ for different length of the magnetized helixes. With the increasing of the rotating frequency, the curvature of the bended helical microswimmers will increase a little both in water and in 12.5% glycerinum. So the distance D will shrink a little with the actuate frequency increasing. Taking LRH4 in water for example, the distance decreases from 7 pixels at 1 Hz to 5 pixels at 3 Hz because of the viscous drag force or torque. We also find another characterized variable called “step-out frequency”, above the step-out frequency leads to poor propulsion performance. Taking LRH4 and LRH2 in water for example, the step-out frequency is 3 Hz and 1.5 Hz respectively in Figure 6b. The reasons are as follows. $v_{forward}$ in water becomes larger than that in 12.5% glycerinum. Due to the larger viscosity of 12.5% glycerinum compared with water, the calculated frequency $f_{act}^{1}$ from (18) may become smaller in 12.5% glycerinum. From Figure 6, the changes D in water and in 12.5% glycerinum is tiny during different actuate frequency $f_{act}$. So the main contributor that leads to smaller $v_{forward}$ in 12.5% glycerinum is the calculated frequency $f_{act}^{1}$ and has nothing to do with the distance D.

**Figure 7:**
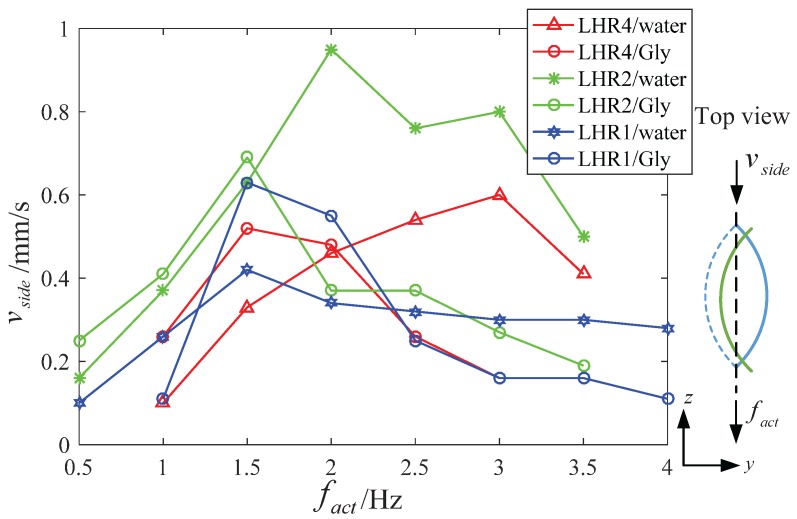
The relation between the propulsion velocity $v_{side}$ and actuate frequency $f_{act}$ for different length.

During spindle-like rotation locomotion on the bottom of the container, a tiny propulsion velocity $v_{side}$ in Figure 7 has been found but its propulsion performance is poor compared with the $v_{forward}$. Firstly, the reason for the propulsion velocity $v_{side}$ is its helical structure during the non-reciprocating motion. Secondly, the reasons for the tiny propulsion velocity $v_{side}$ are the small helix and low actuate frequency $f_{act}$. Another interesting phenomenon is that the same lotus-root microswimmer in 12.5% glycerinum has more larger propulsion velocity $v_{side}$ but more smaller step-out frequency. The reason is that the viscosity in 12.5% glycerinum is larger than that in water. Under the step-out frequency, the experiment results show that the viscosity mainly contributes to the larger propulsion velocity $v_{side}$ in spite of the smaller calculated frequency $f_{act}^{1}$. Above the step-out frequency, the propulsion velocity $v_{side}$ in 12.5% glycerinum drops more heavily than that in water. The experiment results show that the viscosity mainly contributes to the propulsion velocity $v_{side}$ in spite of the bigger calculated frequency $f_{act}^{1}$.

### 4.2. Experiments and Analysis on Spindle-Like Rotation Locomotion with an Inclination Angle

**Figure 8:**
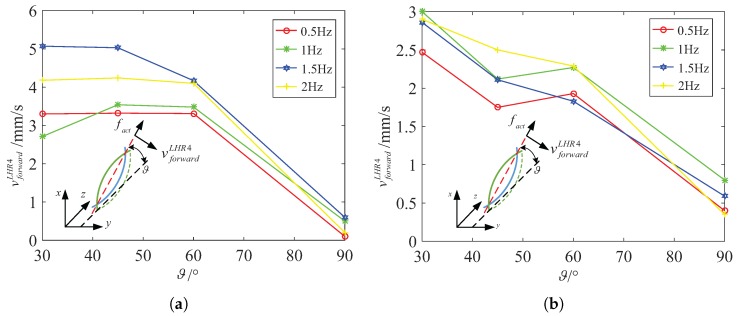
LHR4: the relation between $v_{forward}^{LHR4}$ and the inclination angle ϑ for different actuate frequency $f_{act}$. (**a**) In water (**b**) in 12.5% glycerinum.

In addition, we also focus on another spindle-like rotation locomotion with an inclination angle ϑ and selectively choose $30^{\circ }$, $45^{\circ }$, $60^{\circ }$ and $90^{\circ }$ to calculate the steering vector. We design a series of experiments to show the relation between the inclination angle ϑ and the actuate frequency $f_{act}$. By comparing them, we can find relatively stable and fast swimmer mode rotating along decided axis. Figure 8a describes the functional relation between the swim velocity $v_{forward}^{LHR4}$ and the inclination angle ϑ in water. The spindle-like locomotion velocity $v_{forward}^{LHR4}$ is approximatively linear inverse proportional to the inclination angle ϑ both in water and in 12.5% glycerinum. Figure 8b describes the functional relation between the spindle-like locomotion velocity $v_{forward}^{LHR4}$ and the actuate frequency $f_{act}$ for different inclination angles ϑ in 12.5% glycerinum. It shows that the lotus-root microswimmer locomotes at relative slow velocity compared with that in water. It can be concluded that the higher viscosity leads to lower the spindle-like locomotion velocity $v_{forward}^{LHR4}$.

**Figure 9:**
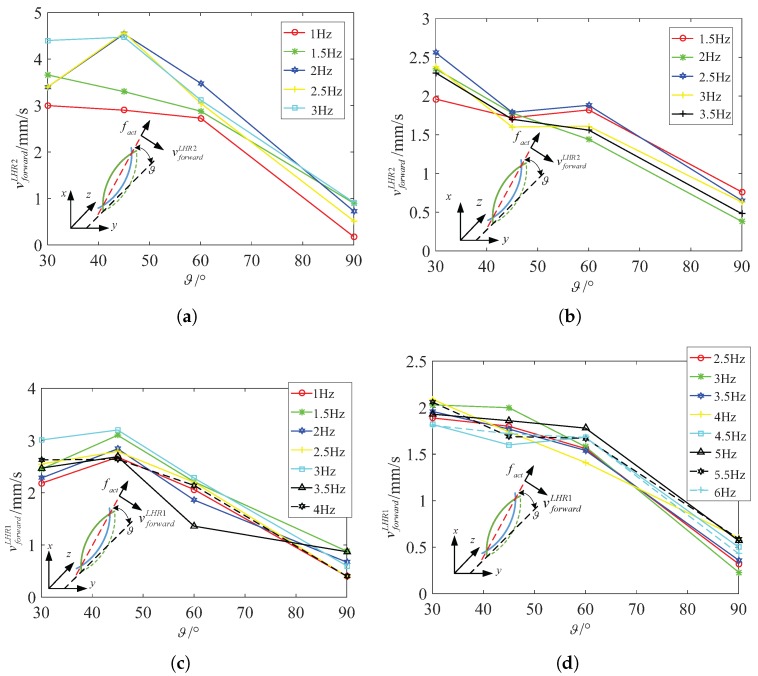
LHR2: the relation between $v_{forward}^{LHR2}$ and the inclination angle for different actuate frequency $f_{act}$. (**a**) In water (**b**) in 12.5% glycerinum; LHR1: the relation between $v_{forward}^{LHR1}$ and the inclination angle ϑ for different actuate frequency $f_{act}$. (**c**) In water (**d**) in 12.5% glycerinum.

Specifically, because of the higher viscosity, the resistance coefficients $C_{n}$ and $C_{l}$ increase which leads to an increase of the denominator of $f_{act}^{3}$ and $f_{act}^{4}$, and an increase of the propulsion force $f_{d}$. Based on analysis of the $f_{c}$ and $f_{d}$, we can make sure that $f_{act}^{3}$ decreases a bit, and that $f_{act}^{4}$ changes a bit. Although $f_{act}^{4}$ may increase or decrease a bit, the experiment results report that the spindle-like locomotion velocity $v_{forward}^{LHR4}$ decreases from in water to in 12.5% glycerinum. So it can be inferred that $f_{act}^{4}$ also decreases and slows down the spindle-like locomotion velocity $v_{forward}^{LHR4}$. In Figure 9, the experiment results verify our above conclusion and show another spindle-like rotation locomotion characteristic. Moreover, LHR2 and LHR1 can keep a higher spindle-like locomotion velocity at $45^{\circ }$ in water but not in 12.5% glycerinum.

**Figure 10:**
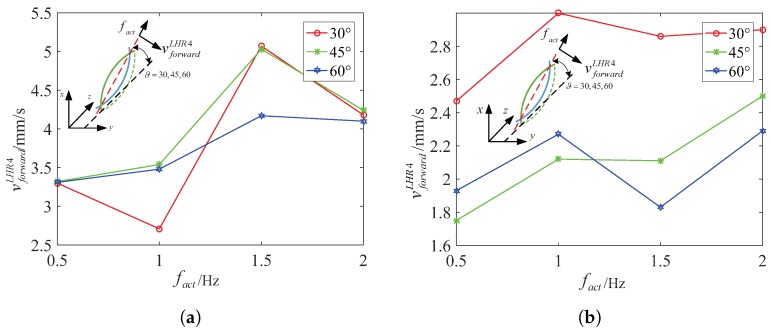
LHR4: the relation between spindle-like rotation locomotion $v_{forward}^{LHR4}$ and actuate frequency $f_{act}$ for different inclination angle ϑ. (**a**) In water (**b**) in 12.5% glycerinum.

**Figure 11:**
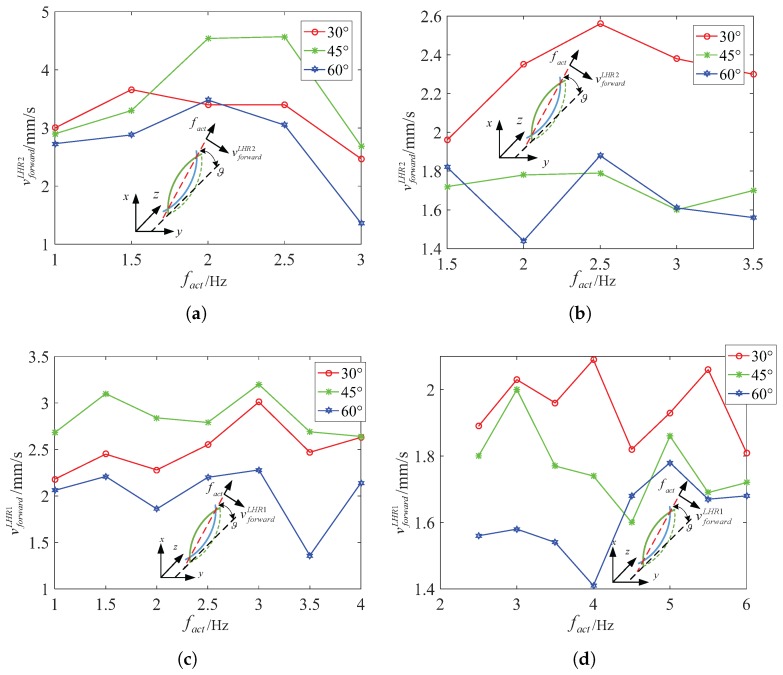
LHR2: the relation between spindle-like locomotion velocity $v_{forward}^{LHR2}$ and actuate frequency $f_{act}$ for different inclination angle. (**a**) In water (**b**) in 12.5% glycerinum; LHR1:The relation between spindle-like rolling velocity $v_{forward}^{LHR1}$ and actuate frequency $f_{act}$ for different inclination angle; (**c**) in water (**d**) in 12.5% glycerinum.

Figure 10 shows that LRH4 in water can locomote at relative high speed at $45^{\circ }$ at four different actuate frequencies, but it can keep stable spindle-like locomotion velocity at $60^{\circ }$ at four different actuate frequencies. In 12.5% glycerinum, it can also keep stable spindle-like locomotion velocity at $60^{\circ }$ but locomote at relative high speed at $30^{\circ }$. In further analysis, during the process of the inclination angle ϑ increasing from $30^{\circ }$ to $90^{\circ }$, $f_{c}$ decreases in water or in 12.5% glycerinum which makes $f_{act}^{4}$ increase a bit. This is approximately in contradiction with the experimental results, but we cannot neglect the parameters $\left(h_{4}-\delta_{4}\right)$. These two parameters jointly change the spindle-like locomotion velocity $v_{forward}^{i}$. Due to the deformability of the lotus root fibers, the distance $\left(h_{4}-\delta_{4}\right)$ between the center and rotation instantaneous axis $p_{axis}$ can change during the spindle-like rotation locomotion. For LHR4, it can be determined that the parameter $\left(h_{4}-\delta_{4}\right)$ reach its maximum at $45^{\circ }$ in water, and at $30^{\circ }$ in 12.5% glycerinum. Thus Figure 11 shows that LHR4 can keep a higher spindle-like locomotion velocity $v_{forward}^{LHR4}$ at $45^{\circ }$ in water, and at $30^{\circ }$ in 12.5% glycerinum. In Figure 11, the experiment results verify our above conclusion for LHR2, LHR1. For LHR2, it can keep relative high locomote velocity $v_{forward}^{LHR2}$ at 2 Hz and 2.5 Hz at $45^{\circ }$ in water, at 1 Hz at $30^{\circ }$ in 12.5% glycerinum. For LHR1, it can keep relative high locomote velocity $v_{forward}^{LHR1}$ at 3 Hz at $45^{\circ }$ in water, at 4 Hz at $30^{\circ }$ in 12.5% glycerinum.

**Figure 12:**
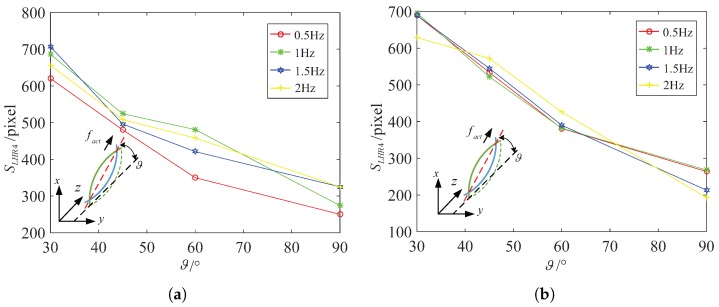
LHR4: the relation between pixel area $S_{LHR4}$ and inclination angle ϑ for different actuate frequency. (**a**) In water (**b**) in 12.5% glycerinum.

Figure 12 describes the functional relation between the area $S_{LHR4}$ of the body and the inclination angle ϑ. According to analysis the little change of the area, we can find that the microswimmer can keep its attitude stable at $45^{\circ }$ in water, at $45^{\circ }$ in 12.5% glycerinum above 0.5 Hz during swimming. The area $S_{LHR4}$ changes between 0.96 mm${}^{2}$ and 1.05 mm${}^{2}$ at $45^{\circ }$ in water, and changes between 1.04 mm${}^{2}$ and 1.14 mm${}^{2}$ at $45^{\circ }$ in 12.5% glycerinum.

**Figure 13:**
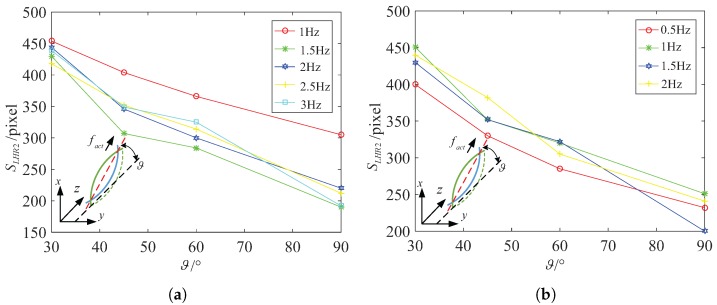

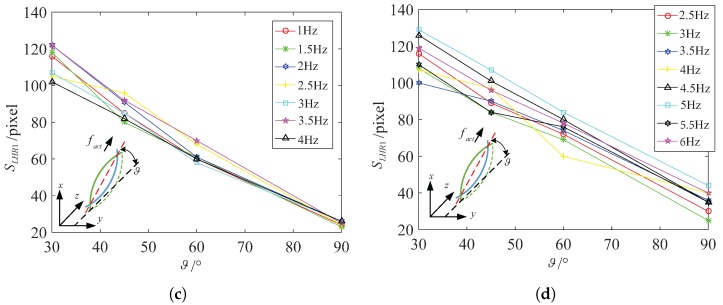
LHR2: the relation between pixel area $S_{LHR2}$ and inclination angle ϑ for different actuate frequency. (**a**) In water (**b**) in 12.5% glycerinum; LHR1: the relation between pixel area $S_{LHR1}$ and inclination angle ϑ for different actuate frequency. (**c**) In water (**d**) in 12.5% glycerinum.

In similar analysis, we can find that LRH2 can keep its attitude stable above 1 Hz during swimming. Particularly, LRH2 can keep its attitude stable at $45^{\circ }$ in water, at $30^{\circ }$ in 12.5% glycerinum above 0.5 Hz during swimming, as shown in Figure 13a,b. The area $S_{LHR2}$ of the helical body fluctuates a little at different inclination angles above 1 Hz. The area $S_{LHR2}$ changes between 0.62 mm${}^{2}$ and 0.71 mm${}^{2}$ at $45^{\circ }$ in water not including 1 Hz, and changes between 0.8 mm${}^{2}$ and 0.9 mm${}^{2}$ at $30^{\circ }$ in 12.5% glycerinum. It is sufficient to verify that LRH2 can both swim at relative high speed $v_{forward}^{LHR2}$ and keep its attitude stable at $45^{\circ }$ at 2 Hz in water, at $30^{\circ }$ at 2 Hz in 12.5% glycerinum.

In Figure 13c,d, we can also find that LRH1 can keep its attitude stable at about 3 Hz during swimming. The area $S_{LHR1}$ changes between 0.116 mm${}^{2}$ and 0.12 mm${}^{2}$ at $60^{\circ }$ in water, and changes between 0.14 mm${}^{2}$ and 0.16 mm${}^{2}$ at $60^{\circ }$ in 12.5% glycerinum. Particularly, LRH1 can keep its attitude stable at $60^{\circ }$ in water or in 12.5% glycerinum.

### 4.3. Discussion

**Figure 14:**
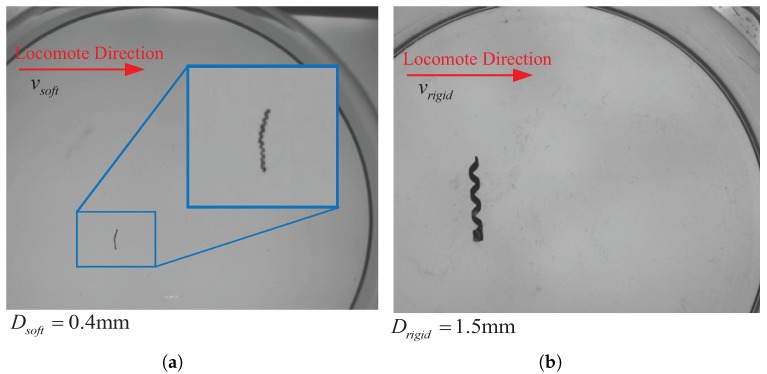
Analysis on propulsion performance at Reynolds number. (**a**) The LHR4 locomotes along the stright line; (**b**) the RHM locomotes along the stright line.The calculated locomotion velocity ratio $\chi_{1}$ decreases to 2.6, so it is confidence that the LHR4 with the soft body can locomote faster.

In this section, we select LHR4 (the left one in Figure 14) and a rigid helical microswimmer (RHM) (the right one in Figure 14) to analysis the propulsion performance of the spindle-like rotation locomotion. As for the RHM, the 60% glycerol solution is selected to simulate the low Reynolds numbers. Its density and viscosity in 20 ${}^{\circ }$C are 1.14 g/cm${}^{3}$ and 60 mPa·s, respectively. The locomotion velocity of the helical swimmer is 6–13 mm/s. Therefore, the calculated Reynolds number is approximately 0.17–0.41 which is similar to the low Reynolds number 0.16–0.55 for LHR4. The measured diameter $D_{soft}$ of the helix of the LHR4 is about 0.4 mm and the measured diameter $D_{rigid}$ of the helix of RHM is about 1.5 mm. If the body of LHR4 is rigid, the theoretical locomotion velocity ratio χ1=vrigidvrigidvsoftvsoft is 3.75 with the same actuate frequency. Nevertheless, the calculated locomotion velocity ratio $\chi_{1}$ is 2.6. Therefore, it is confidence that the LHR4 with the soft body can locomote faster.

Due to the heterogeneous distribution of the shrinking stress, the soft helical microswimmers are bent lightely initially for different length. In addition, due to their soft and deformable bodies, they undergo a swimming motion which is a spindle-like rotation locomotion. The propulsion mechanism of the spindle-like rotation locomotion change the equivalent rotation radius from the radius $D_{soft}/2$ to the bigger one $\delta_{1}$ or $\delta_{2}$ (see Figure 2b,d). Consequently, the propulsion performance of spindle-like rotation locomotion is better than helical propulsion.

The guideline to make a faster microswimmer is to find out the optimized curvature-length ratio χ2=DDLL. In this paper, three helical microswimmers with different length including 4 mm, 2.3 mm, 1.4 mm, named LRH4, LRH2 and LRH1 are used in Figure 5c. From Figure 6a, the curvature-length ratio $\chi_{LHR4}\approx 1.25$, $\chi_{LHR2}\approx 1.3$ and $\chi_{LHR1}\approx 1.42$ can be calculated when actuate frequency $f_{act}>$ 1.5 Hz. From Figure 6b, we can LRH2 locomotes faster than others both in water and 12.5% glycerinum when actuate frequency $f_{act}>1.5$ Hz. To obtain better propulsion performance of the fabricated microswimmers, it is better to keep the curvature-length ratio $\chi_{2}$ within 1.25 and 1.42.

## 5. Conclusions

In conclusion, we present a lotus-root-based magnetically propelled helical microswimmer, which is fabricated from the fiber of lotus-root coated with magnetic nanoparticles. This fabrication method is easy to achieve at low cost. The new helical microswimmers are fabricated by the simple coating of lotus-root-based fibers with a thin magnetic layer. As the fiber of lotus-root with magnetic coating is deformable, the microswimmers show an attracting spindle-like rotation locomotion actuated by the uniform rotate magnetic field. Spindle-like rotation locomotion in water or in 12.5% glycerinum is analyzed in detail. By modeling its dynamical model in spindle-like rotation locomotion and spindle-like rotation locomotion with an inclination angle, we have established the relation between the spindle-like locomotion velocity and the actuate frequency respectively. we find out that the curvature of the shape decreases as a function of actuate frequency, which is related to the deformability of lotus-root fibers, and that the microswimmer can locomote faster than that with rigid body in both experiments actuated by uniform rotate magnetic field. In addition, the viscosity is proved to have a great impact on the spindle-like locomotion velocity. Specifically, the higher the viscosity is in 12.5% glycerinum, the lower $v_{forward}$ is in spindle-like locomotion and spindle-like locomotion with an inclination angle, but the higher $v_{side}$ becomes in spindle-like locomotion. In the future, a closed-loop visual servo control algorithm will be employed and the steer characteristics will be analyzed in spindle-like rotation locomotion and spindle-like rotation locomotion with an inclination angle.
